# Characterization and Recombinant Expression of Terebrid Venom Peptide from *Terebra guttata*

**DOI:** 10.3390/toxins8030063

**Published:** 2016-03-03

**Authors:** John Moon, Juliette Gorson, Mary Elizabeth Wright, Laurel Yee, Samer Khawaja, Hye Young Shin, Yasmine Karma, Rajeeva Lochan Musunri, Michelle Yun, Mande Holford

**Affiliations:** 1 Hunter College, City University of New York, Belfer Research Center 413 E. 69th Street, New York, NY 10021, USA; John.jj.moon@gmail.com (J.M.); jgorson@gradcenter.cuny.edu (J.G.); lzwright@gmail.com (M.E.W.); yeelaurel93@gmail.com (L.Y.); samerk28@gmail.com (S.K.); hyshin1209@gmail.com (H.Y.S.); yrkarma@gmail.com (Y.K.); yunm11@gmail.com (M.Y.); 2 The Graduate Center, City University of New York, 365 Fifth Avenue, New York, NY 10016, USA; 3 New York Genome Center, 101 Avenue of the Americas, New York, NY 10013, USA; raj.musunuri@gmail.com; 4 The American Museum of Natural History, 79th Street at Central Park West, New York, NY 10026, USA

**Keywords:** Terebridae, venom peptides, teretoxins, recombinant synthesis, polychaete assay, disulfide-rich peptides, Conoidea, snail venom

## Abstract

Venom peptides found in terebrid snails expand the toolbox of active compounds that can be applied to investigate cellular physiology and can be further developed as future therapeutics. However, unlike other predatory organisms, such as snakes, terebrids produce very small quantities of venom, making it difficult to obtain sufficient amounts for biochemical characterization. Here, we describe the first recombinant expression and characterization of terebrid peptide, teretoxin Tgu6.1, from *Terebra guttata*. Tgu6.1 is a novel forty-four amino acid teretoxin peptide with a VI/VII cysteine framework (C–C–CC–C–C) similar to O, M and I conotoxin superfamilies. A ligation-independent cloning strategy with an ompT protease deficient strain of *E. coli* was employed to recombinantly produce Tgu6.1. Thioredoxin was introduced in the plasmid to combat disulfide folding and solubility issues. Specifically Histidine-6 tag and Ni-NTA affinity chromatography were applied as a purification method, and enterokinase was used as a specific cleavage protease to effectively produce high yields of folded Tgu6.1 without extra residues to the primary sequence. The recombinantly-expressed Tgu6.1 peptide was bioactive, displaying a paralytic effect when injected into a *Nereis virens* polychaete bioassay. The recombinant strategy described to express Tgu6.1 can be applied to produce high yields of other disulfide-rich peptides.

## 1. Introduction

**Figure 1:**
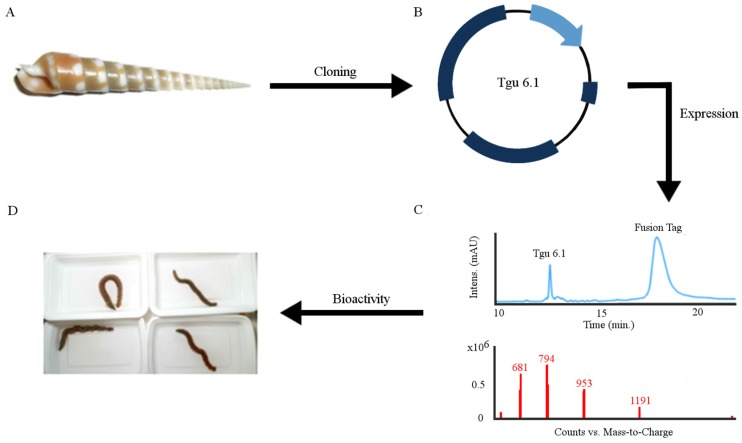
Recombinant expression and characterization of Tgu6.1 teretoxin. (**A**) Terebrid snail *Terebra guttata* from which the Tgu6.1 teretoxin was discovered. (**B**) Plasmid map of Tgu6.1 cloned into pET-32a Xa/ligation independent cloning (LIC) vector via LIC. (**C**) RP-HPLC purification of recombinant Tgu6.1 from its fusion tag after expression, purification and cleavage (top spectra); LC-MS analysis of Tgu6.1 (bottom spectra). (**D**) Characterization of Tgu6.1 bioactivity using the native prey polychaete worm assay (view the video in Supplemental Material).

Venom peptides are a resource for investigating evolution, cellular communication and therapeutic development [1,2,3]. The Terebridae are a family of venomous marine gastropods in the superfamily Conoidea, which includes cone snails (family Conidae) and turrids (a complex family) [4,5,6]. Like most conoideans, some terebrid species produce venoms that consist of greater than 100 different peptides that are primarily used to capture prey [7,8,9,10,11]. Research of conoideans over the last three decades largely focused on the venom peptides of cone snails (conotoxins) [11,12,13]. Collective biochemical and pharmacological evidence has shown that conotoxins are both diverse and highly selective, binding specifically to various ligand-gated and voltage-gated ion channel subtypes, including sodium, potassium and calcium channels, and can be used as molecular probes and pharmaceutical agents [10,14,15,16]. In contrast, the toxicology of terebrids has not yet attracted the same degree of scientific attention. In large part, this is because terebrids are smaller and produce venom on the nanogram scale. However, with technological advances, such as DNA and RNA sequencing, PCR and molecular biology, it is now possible to investigate the primary sequences of terebrid venom peptides, teretoxins, using an integrated venomics strategy that combines phylogenetics, transcriptomics and proteomics [8,9,17,18]. Here, we describe the characterization of a novel teretoxin, Tgu6.1 from *Terebra guttata* (Figure 1).

Teretoxins and conotoxins, while similar in molecular structure, are not homologous. Teretoxins differ from conotoxins in size, complexity and structural integrity, suggesting possible diverse molecular applications [7,19,20]. Recently, mature teretoxins from *Terebra subulata* and *Hastula hectica* were identified with cysteine frameworks similar to those found in conotoxins, but without signal sequence homology, suggesting divergence in the venom peptide evolution [20]. Additionally, recent isolation and structural characterization of teretoxin Tv1 from *Terebra* *variegata* identified Tv1 as similar to M-superfamily conotoxins; however, Tv1 has a unique fold and disulfide-bonding pattern not previously found in venom peptides [2]. These findings suggest that teretoxins are a promising resource to increase the venom peptide toolbox.

Despite the potential of terebrid venom peptides, given the small size of terebrid snails, obtaining sufficient amounts of venom for downstream biochemical characterization remains a significant challenge. After obtaining the primary sequence of venom peptides, solid phase peptide synthesis (SPPS) and recombinant expression are often applied to produce synthetic versions of the peptides for investigation [21,22,23]. SPPS has the advantage of being able to incorporate unnatural amino acids and accommodating posttranslational modifications. Traditionally, SPPS was restricted to direct synthesis to peptides of <50 amino acids; however, modern advances, such as microwave synthesis and peptide ligation methods, have extended the size of peptides made synthetically significantly to <100 amino acids [24,25,26,27,28]. Teretoxins are generally larger than conotoxins, ranging in size up to 70 amino acids, which places them at the upper limits of SPPS and, therefore, are appealing candidates for recombinant expression. There are several examples in the literature of the recombinant expression of short disulfide-rich peptides [23,29,30,31,32,33,34,35,36,37,38,39]. Table 1 highlights the different aspects of the recombinant expression that must be considered, such as the choice of fusion tag, purification method, host species and strain and method of cleavage. In this study, we outline a strategy for recombinantly-expressing teretoxins with the first successful recombinant expression and purification of teretoxin Tgu6.1 (Figure 1). Teretoxin Tgu6.1 is a novel forty-four amino acid peptide from *Terebra* *guttata* with a VI/VII cysteine framework (C–C–CC–C–C), which can also be found in the conotoxin M-, I- and O-superfamilies [40,41,42,43,44]. The bioactivity of Tgu6.1 was also characterized using a bioassay of native prey *Nereis virens* polychaete worms. The recombinant strategy outlined can be readily applied to other teretoxins and disulfide-rich peptides.

## 2. Results and Discussion

### 2.1. Design and Construction of Teretoxin Tgu6.1 Expression System

**Figure 2:**
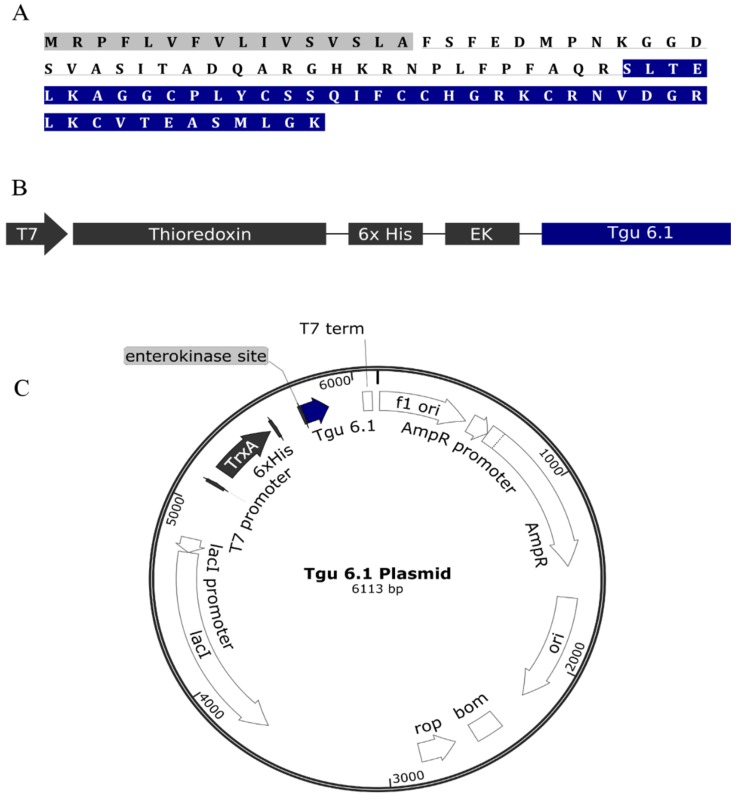
Recombinant expression strategy of Tgu6.1. (**A**) Full precursor structure of Tgu6.1. The signal sequence is shaded in gray; the pro-region is underlined; and the mature peptide is shaded in blue. (**B**) Schematic representation of Tgu6.1 fusion protein. The fusion protein was expressed under the control of a pET-32a T7 promoter and contains thioredoxin as the fusion partner, His6-tag for purification and the enterokinase (EK) site for the cleavage of Tgu6.1 from TRX by enterokinase. (**C**) Plasmid map of the expression vector. The Tgu6.1 gene was cloned into pET-32a XA/LIC plasmid by ligation independent cloning.

Tgu6.1 was obtained and identified on the genetic level as its full precursor sequence using RNA-Seq (Figure 2A). The mature peptide Tgu6.1 was expressed as a fusion protein sequentially composed of an N-terminal thioredoxin tag, Histidine-6 (His6) tag and enterokinase (EK) site (Figure 2B). Small cysteine-rich peptides are difficult to overexpress in *Escherichia coli* due to the formation of insoluble protein aggregates or inclusion bodies, proteolytic degradation and reducing conditions in the *E. coli* cytoplasm that hinder the formation of disulfide bonds [14]. Several choices were made in the design of the expression system of Tgu6.1 to mitigate these issues.

Specifically, solubility was addressed by the cloning of the Tgu6.1 gene into thioredoxin containing pET-32a XA/LIC vector using ligation independent cloning (LIC) (Figure 2C). Thioredoxin as a fusion partner has been shown to significantly increase the solubility of proteins synthesized in the *E. coli* cytoplasm [30,45,46,47,48]. Furthermore, when expressed in an oxidizing environment, thioredoxin has been observed to catalyze disulfide bond formation [49]. Proteolytic degradation was attenuated by expressing the fusion protein in an ompT protease-deficient *E. coli* strain, Origami B (DE3). This strain also provides the oxidizing cytoplasmic environment necessary for disulfide bond formation via its double (trxB^−^, gor^−^) mutation [50]. To further optimize expression, the Tgu6.1 gene was reverse translated from the amino acid sequence to codons optimized for *E. coli* bacterial expression. A His6-tag was provided by the pET-32a Xa/LIC vector for purification of the fusion protein via immobilized metal affinity chromatography. Finally, an enterokinase site was added directly to the N-terminus of Tgu6.1 allowing for fusion peptide cleavage without excess residues on the cleaved Tgu6.1 peptide product.

### 2.2. Expression and Purification of Tgu6.1

**Figure 3:**
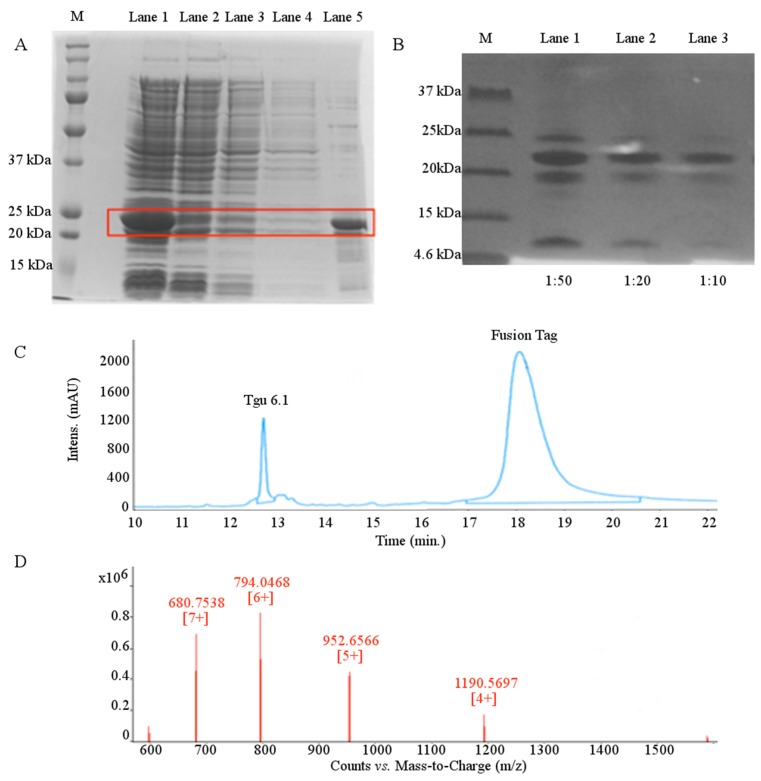
Expression and purification of Tgu6.1. (**A**) 12% SDS-PAGE Coomassie-stained gel showing expression and purification of Tgu6.1 fusion protein by Ni-NTA affinity chromatography. M = protein molecular weight marker; Lane 1 = cell lysate; Lane 2 = supernatant post-binding to Ni-NTA resin; Lane 3 = Wash Buffer 1 supernatant; Lane 4 = Wash Buffer 2 supernatant; Lane 5 = imidazole eluted fraction. (**B**) Tris-tricine 16.5% SDS-PAGE Coomassie-stained gel showing Tgu6.1 cleavage by enterokinase. M = protein molecular weight marker; Lanes 1–3, enterokinase cleavage in 1:50, 1:20 and 1:10 dilutions. (**C**) Chromatogram of RP-HPLC purification of Tgu6.1 from TRX fusion tag. An X-Bridge C18 semi-preparative column was used with Buffer A (0.1% TFA) and Buffer B (80% ACN/0.1% TFA). The peptide was eluted with a linear gradient of 5%–75% Buffer B over 30 min at a flow rate of 5 mL/min. (**D**) LC-MS characterization of folded Tgu6.1. The +4, +5, +6 and +7 ion charge states are shown. Expected mass = 4758.58 Da. Observed mass = 4758.28 Da.

Expression of Tgu6.1 fusion protein was induced by addition of 0.4 mM isopropyl β-d-1-thiogalactopyranoside (IPTG) during log phase growth, at 25 °C. After overnight expression and subsequent lysis by sonication, the fusion protein was purified from the soluble fraction by Ni-NTA (Nickel-NitriloTriacetic Acid) affinity chromatography batch-wise and under native conditions. The 23.77-kDa fusion protein was eluted with 500 mM imidazole, and its overexpression and purity were confirmed with SDS-PAGE (Figure 3A).

Following ultrafiltration and buffer exchange, the fusion protein was cleaved with recombinant enterokinase. Cleavage was observed by Tris-tricine SDS-PAGE with the appearance of two bands at 20 kDa and 4.8 kDa, corresponding to the fusion tag and cleaved Tgu6.1, respectively (Figure 3B). Cleavage conditions were optimized with the addition of urea, as inaccessibility to the cleavage site has been shown to favor advantageous non-specific cleavage [51]. Partial denaturation by urea both improved the yield of cleaved Tgu6.1 and reduced non-specific cleavage significantly.

Fusion free Tgu6.1 was then purified and collected by reverse-phase HPLC (RP-HPLC). Two major peaks were observed at 12.7 and 18 min (Figure 3C). Liquid chromatography mass spectrometry (LC-MS) confirmed that the two peaks observed were the oxidized Tgu6.1 and the TRX fusion tag, respectively. The 12.7-min peak corresponding to the oxidized Tgu6.1 displayed a monoisotopic mass of 1190.57 *m/*z, which is the M + 4H charge corresponding to a mass of 4758.28 Da, which is consistent with the predicted mass of 4758.58 Da for fully-oxidized Tgu6.1. The M + 5H, M + 6H and M + 7H charged states were also observed (Figure 3D). Expression of Tgu6.1 in the Origami host gave an average yield of 20.9 mg per liter of growth medium.

### 2.3. Polychaete Functional Assay

The bioactivity of Tgu6.1 was examined using a *Nereis* *virens* polychaete bioassay. Polychaetes, such as *N. virens*, are the natural prey of terebrid snails and were previously shown to be a viable assay for determining the bioactivity of teretoxins [9,19]. The polychaete bioassay provides a global phenotypic detection of teretoxin bioactivity by observing the behavioral response of the worm to treatment with the peptide. Polychaetes are also readily available in laboratory cultures and also widely used to examine the toxicity of organic chemicals [51].

**Figure 4:**
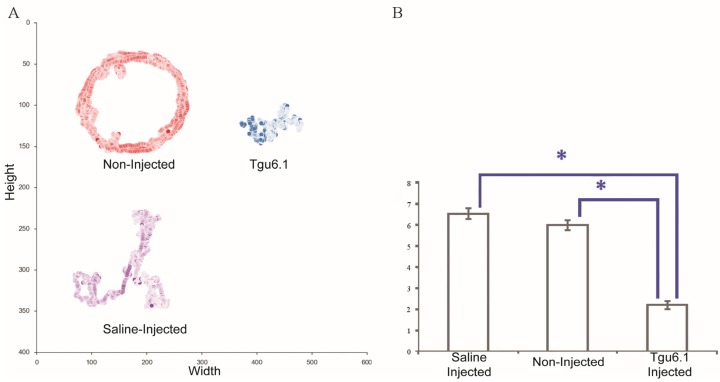
Representative centromeres and average moving speed. (**A**) Visualization of the movement of the polychaetes for ten minutes after injection. (**B**) Graph depicting the mean and standard error of the average speeds. The * represents *p*-values less than 0.05.

Under standardized conditions, 10 µmoles/g of novel synthetic teretoxin Tgu6.1 were injected into the central nerve cord of each polychaete worm (Figure 4). Polychaetes injected with Tgu6.1 resulted in a significantly lower average moving speed than non-injected (2.189 ± 0.199 *vs.* 5.975 ± 0.225; *df* = 2, F = 107.6; *p*-value < 0.0001) and saline-injected worms (2.189 ± 0.199 *vs.* 6.526 ± 0.256; *p*-value < 0.0001). Saline-injected and non-injected worms did not show significance (*p*-value of 0.201). These findings indicate that there is strong evidence that Tgu6.1 caused a paralytic effect in *N.* *virens*.

## 3. Experimental Section

### 3.1. Construction of Recombinant Plasmid

The Tgu6.1 mature peptide sequence obtained from RNASeq (GenBank Accession Number KU738608) was optimized for *E. coli* codon usage. The synthetic Tgu6.1 insert was ordered from Integrated DNA Technologies (IDTDNA). The gene insert was amplified using primers 5′-GGTATTGAGGGTCGCATATTATATTATTTA-3′ and 5′-AGAGGAGAGTTAGAGCCATAATAATATTTA-3′ (IDTDNA), which contained the requisite ligation-independent cloning (LIC); overhangs underlined in the sequence above.

The PCR amplified insert was purified with SpinPrep Gel DNA Kit (Novagen, Darmstadt, Germany) and cloned via LIC into vector pET-32 Xa/LIC (EMD Millipore, Darmstadt, Germany). The insert was treated for 30 min at 22 °C with T4 DNA polymerase at 0.5 unit per 0.1 pmol/µL of insert in TlowE (Tris-low- EthyleneDiamineTetraAcetic acid (EDTA)) buffer (10 mM Tris HCl, 0.1 mM EDTA, pH 8.0) with 2.5 mM dGTP and 5 mM DTT. The enzyme was inactivated at 75 °C for 20 min. The T4 DNA polymerase-treated insert was annealed into the Xa/LIC vector at 22 °C for 5 min. Then, 7.25 mM EDTA was added, and the components were stirred with a pipet tip for another 5 min at 22 °C. The pET-32 Xa/LIC:Tgu6.1 plasmid construct was transformed into *E. coli* NovaBlue obtained from Novagen. Positive clones were screened for via ampicillin and kanamycin resistance. Insertion was verified by colony PCR (EMD Millipore) and DNA sequencing. For colony PCR, single colonies of screened positive clones were suspended in 50 µL of water, incubated at 99 °C for 5 min and centrifuged at 12,000× *g* for 1 min. Ten microliters of supernatant were used for PCR using the T7 promoter and the T7 terminator (IDTDNA) as the forward and reverse primers, respectively.

### 3.2. Induction and Expression

The pET-32 Xa/LIC:Tgu6.1 plasmid construct verified by colony PCR and DNA sequencing was transformed into *E. coli* Origami (Novagen) for expression. A single colony from a fresh plate was used to inoculate a primary culture of LB media containing tetracycline (12.5 μg/mL) and ampicillin (50 μg/mL). The primary culture was grown overnight at 37 °C with shaking at 250 RPM. A larger culture was inoculated using the overnight pre-culture. The cells were incubated at 37 °C and 250 RPM until the OD_600_ was between 0.8 and 1.0. Isopropyl β-d-1-thiogalactopyranoside was then added to a final concentration of 0.4 mM to induce the expression of the fusion protein. The culture was incubated overnight at 25 °C and 250 RPM for overexpression of soluble protein. The cells were harvested by centrifugation (8000× *g*, 10 min, 4 °C), and the pellet was stored at −20 °C until use.

### 3.3. Protein Extraction and His-Tag Affinity Purification

The bacterial pellet of *E. coli* Origami transformed with the pET-32 Xa/LIC:Tgu6.1 plasmid construct was resuspended in sodium phosphate buffer (100 mM Na-PO4, pH 8.0, 300 mM NaCl, 10% glycerol) and lysed by sonication using Fisher Scientific (Hampton, VA, USA) Model 120 Sonic Dismembrator with three rounds at 70% power for 30 s and three rounds at 90% power for 30 s for soluble protein extraction. Cleared lysate was generated by centrifugation at 13,000× *g* for 45 min.

The supernatant was purified with a batch purification method using nickel-NTA resin (Qiagen, Hilden, Germany) pre-equilibrated with lysis buffer (100 mM Na-PO4, 300 mM NaCl, 10% glycerol, 1 mg/mL lysozyme, pH 8.0). Binding of cleared fusion protein lysate to nickel-NTA resin was followed by treatment with two wash buffers: Wash Buffer 1 (50 mM Na-PO4 pH 7.7, 300 mM NaCl, 10 mM imidazole, 10% glycerol) and Wash Buffer 2 (50 mM NaPO4 pH 7.7, 2M NaCl, 10 mm imidazole, 10% glycerol). His-tagged protein was eluted sequentially with two elution buffers, Elution Buffer 1 (50 mM Na-PO4 pH 7.7, 300 mM NaCl, 20 mM imidazole) and Elution Buffer 2 (50 mM Na-PO4 pH 7.7, 300 mM NaCl, 500 mM imidazole, 10% glycerol). Fusion protein was desalted by buffer exchange to 1× phosphate buffer saline (PBS) via ultrafiltration (10 kDa MWCO, Sigma-Aldrich, St. Louis, MO, USA). Expression and purification were analyzed by SDS-PAGE (Bio-Rad Laboratories, Hercules, CA, USA).

### 3.4. Enterokinase Cleavage

The purified fusion protein was cleaved by enterokinase protease (EMD Millipore and Syd Labs). Cleavage was incubated overnight at an enzyme:substrate ratio of 1:50 in EK cleavage capture buffer (50 mM NaCl, 20 mM Tris-HCl, 2 mM CaCl_2_, pH 7.4). Cleavage yield was enhanced by the addition of urea to a final concentration of 1 to 4 M; optimal cleavage was observed at a final concentration of 3 M urea.

### 3.5. RP-HPLC Purification and Mass Spectrometry

Cleaved Tgu6.1 was purified by RP-HPLC (Agilent, Santa Clara, CA, USA) using an X-Bridge C18 semi-preparative column (10 × 150 mm, 5-µm particle size, Waters Corporation, Milford, MA, USA) pre-equilibrated with 95% Buffer A (0.1% TFA). Elution was carried out at 5 mL/min over a linear gradient of Buffer B (80% acetonitrile 0.1% TFA) from 5% to 75% in 30 min. ESI-mass spectra were recorded on an Agilent Technologies 6520 Accurate-Mass Q-TOF LC/MS. Samples were delivered to the mass spectrometer through chromatographic separation on the Agilent HPLC 1290, and monoisotopic average masses of peptides were calculated from sequence information using the UCSF ProteinProspector MS-Product tool ([52], San Francisco, CA, USA). Observed mass was calculated from *m*/*z* charged states using MassHunter Bioconfirm Qual B.06 software (Agilent, Santa Clara, CA, USA, 2012).

### 3.6. Polychaete Worm Assay

A *Nereis virens* polychaete bioassay was used to examine the bioactivity of Tgu6.1. For benchmark trials of each experiment, three *N. virens* were placed in cold saltwater solution (4 °C), with a 20-min acclimation period, preceding the injection. The acclimation period accustomed polychaetes to their experimental environment, thus shifting the worms from salt-water solution (4 °C) to room temperature (25 °C), stimulating the phenotypic response. Worms used were comparable in size (<9.0 g, <9 cm) to ensure consistency of activity upon teretoxin injection. Control worms were injected with 2 µL of substance per 2 g of normal saline solution (NSS). Tgu6.1 teretoxin was dissolved in NSS and diluted to 20 µM aliquots. Three worms were used for each experimental condition: non-injected, saline solution and Tgu6.1; and the experiment was repeated in triplicate. Worms were injected with teretoxin using B-D (Becton Dickinson, Franklin Lakes, NJ, USA) ½ cc LO-DOSE U-100 insulin plastic syringe 28 G 1/2 (0.36 mm × 13 mm) with altered needle caps, to manage a uniform 1.0-mm depth of needle puncture. To target the ventral nerve cord, subjects were injected between the 5th and 7th segment of the ventral anterior end. Phenotypic worm movement and behavior were recorded on video for a duration of 2.5 h and were used to assess the effects of each teretoxin injection. Post-injection, polychaetes showed excitatory movement at room temperature, whereas injections with salt-water solution had a sedating result. Changing temperatures distinguished the variability in overall spatial range of movement and the average speed of the polychaetes. The first 10 min after each teretoxin Tgu 6.1 injection resulted in partial paralysis of the polychaete worm.

Video recordings of each polychaete injection were analyzed frame-by-frame using an in-house custom image segmentation algorithm to accurately separate worm contours from the underlying pixel noise. The algorithm was implemented in Python using two open source computer vision libraries: OpenCV and SimpleCV. The algorithm begins with a palletization function based on k-means clustering to minimize the number of image segments expected to be seen in the video streams with worm data. This step corrects for inaccuracies in the image segments in frames with high noise. The video stream-specific palette generated is then used to segment each image frame into multiple contours. The worm contours are obtained by filtering out the non-worm contours by size, color and position. The mass weighted centroids are then calculated for each worm contour obtained. This is then further utilized to obtain per-frame worm centroid movement speed. The pseudocode for the image segmentation algorithm used is shown below.

Pseudocode describing in-house image segmentation algorithm to analyze polychaete movement:

        randomFrame ≤ videoStream
        FUNCTION LearnPalette(randomFrame, [binSize1, binSize2, ..., binSizeN])
             RETURN optimalNumberOfImageSegments, learnedPalette
        ENDFUNCTION
        FOR frame ≤ videoStreamStart TO videoStreamEnd
              FUNCTION FindCountoursWithPalette(frame, learnedPalette)
                   IF foundCountours > optimalNumberOfImageSegments THEN
                          newOptimalNumberOfImageSegments, newPalette = LearnPalette(frame, [binSize1, binSize2, ..., binSizeN])
                          newContours = FindContoursWithPalette(frame, newPalette)
                          RETURN newContours  
                   ELSE
                          RETURN foundContours
                   ENDIF
              ENDFUNCTION
              FUNCTION GetWormCountours(foundCountours)
                   wormContours = SizeAndBoundingBoxFilter(foundContours)
				   RETURN wormContours
              ENDFUNCTION
              FUNCTION GetMassWeightedCentroids(wormContours)
                   RETURN wormCentroids
              ENDFUNCTION
        ENDFOR
		

## 4. Conclusions

Venom peptides from terebrid snails increase the toolbox of bioactive compounds that can be used to characterize cellular communication and potentially applied to the development of therapeutics for improving human health. Unlike snakes, terebrid snails produce nanogram quantities of venom, making it difficult to obtain sufficient amounts for biochemical characterization. Here, we describe the first recombinant expression of a teretoxin and characterize the bioactivity of novel Tgu6.1 teretoxin from *Terebra guttata.*

To produce Tgu6.1 recombinantly, a ligation independent cloning strategy with an ompT protease-deficient strain of *E. coli* as a vector to express Tgu6.1 was employed. Several considerations in plasmid design where made to combat common challenges associated with recombinant expression, such as the formation of insoluble protein aggregates in *E. coli*, proteolytic degradation and unfavorable conditions in *E. coli* cytoplasm that can prevent the formation of disulfide bonds. Recombinant expression of Tgu6.1 provided an average yield of 20.9 mg per liter of growth medium to apply for bioactivity assays (Figure 1). This carefully constructed recombinant design can be used as an alternative to solid phase peptide synthesis of teretoxins and other disulfide-rich peptides. As research in venom peptides for therapeutic drug development increases, it is crucial to have reliable methods for obtaining significant amounts of disulfide-rich peptides.

While Tgu6.1 requires further characterization to identify its specific molecular target, we have successfully demonstrated its paralytic activity in an *N. virens* polychaete bioassay. Prior research of teretoxin Tv1 from *Terebra variegata* also produced paralytic activity in a similar polychaete assay, and characterization of crude terebrid venom extract from several species suggests that a possible molecular target for teretoxins could be nicotinic receptors [8,17,18]. Taken together, the results from this work demonstrate that teretoxins are promising venom peptides that can be recombinantly expressed for biochemical characterization.

## Figures and Tables

**Table 1:** Comparison of recombinant expression strategies for disulfide-rich peptides. Main features of recombinant expression systems for venom peptides from cone snails, scorpion, sea anemone and spider.

Organism	Peptide Features	N-terminal Fusion Tag	Purification Method	Soluble/Insoluble	*E. coli* Host	Fusion Tag Cleavage	Reference
MVIIA (Conus magus)	25 aa and 3 disulfide	Thioredoxin	His tag	Soluble	BL21 (DE3)	No cleavage	Zhan *et al*., 2003 [29]
BgK (Bunodosoma granulifera)	37 aa and 3 disulfide	S-tag (C-terminal)	S-protein	Soluble	OrigamiB (DE3) Tuner (DE3)	No cleavage	Braud *et al*., 2004 [30]
Mo1659 (Conus monile)	13 aa and no cysteine	Cytochrome-b5	His tag	Soluble	Bl21 (DE3)	CnBr	Kumar *et al*., 2005 [31]
Conkunitzin-S1 (Conus striatus)	60 aa and 2 disulfide	Chitin binding domain	His tag	Insoluble	BL21 (DE3)	Intein	Bayrhuber *et al*., 2006 [32]
Lt7a (Conus litteratus)	27 aa and 3 disulfide	Thioredoxin	His tag	Soluble	BL21 (DE3)	Factor Xa	Pi *et al*., 2006 [33]
BTK-2 (Mesobuthus tamulus)	32 aa and 3 disulfide	Cytochrome-b5	His tag	Soluble	BL21 (DE3)	Tev protease	Kumar *et al*., 2009 [34]
Vn2 (Conus ventricosus)	33 aa and 3 disulfide	Glutathione-S-transferase	His tag	Soluble	BL21 (DE3)	No cleavage	Spiezia *et al*., 2012 [35]
PrIIIE (Conus parius)	22 aa and 3 disulfide	Small ubiquitin-like modifier (SUMO)	His tag	Soluble	Rosetta-gami B (DE3)	SUMO protease	Hernandez *et al*., 2012 [36]
MrVIB (Conus marmoreus)	31 aa and 3 disulfide	Pe1B leader signal peptide	His tag	Soluble	BL21 (DE3)	No His tag cleavage	Gao *et al*., 2013 [37]
Huwenotoxin-IV (Ornithoctunus huwena)	35 aa and 3 disulfide	SUMO	His tag	Soluble	Shuffle T7 Express	SUMO protease	Sermadiras *et al*., 2013 [38]
spider, sea anemone, scorpion, cone snail, centipede	Varied length disulfide-rich peptides	Various	various	Soluble	BL21 (DE3)	various	Klint *et al*., 2013 [23]
Framework XV conotoxins (various species)	Varied length and 4 disulfide	Thioredoxin	His tag	Soluble	BL21 (DE3)	Enterokinase	Wu *et al*., 2014 [39]

## Supplementary Materials

The following are available online at www.mdpi.com/2072-6651/8/3/63/s1.
